# Prostate Cancer Care in Rural Primary Care Contexts: A Narrative Review

**DOI:** 10.7759/cureus.68890

**Published:** 2024-09-07

**Authors:** Ryuichi Ohta, Chiaki Sano

**Affiliations:** 1 Community Care, Unnan City Hospital, Unnan, JPN; 2 Community Medicine Management, Shimane University Faculty of Medicine, Izumo, JPN

**Keywords:** cultural sensitivity, disparity, general medicine, healthcare disparities, primary care, prostate cancer, psa testing, rural healthcare, telehealth

## Abstract

Prostate cancer is highly prevalent among older men and poses significant health challenges, particularly in rural areas where access to specialized care is limited. This narrative review aims to evaluate the quality of prostate cancer care in rural primary care settings, identify gaps, and suggest strategies for improvement. A comprehensive narrative review was conducted using PubMed to identify relevant studies published between April 2000 and August 2024. The search focused on articles discussing prostate cancer management in rural primary care, including challenges, outcomes, and collaborative practices. Thirteen studies met the inclusion criteria and were analyzed to assess the quality of care and potential areas for enhancement. The review highlighted significant disparities in prostate cancer care in rural areas, including limited access to urologists, variability in PSA testing practices, and socioeconomic and geographic barriers. Innovative models like telehealth and 'One Stop' Prostate Clinics (OSPCs) showed promise in addressing these challenges. However, gaps in long-term symptom management and follow-up care persist, emphasizing the need for comprehensive survivorship plans and targeted educational interventions for primary care physicians. Rural primary care settings face unique challenges in managing prostate cancer, necessitating tailored strategies to improve care quality. Enhancing collaboration between primary care physicians and urologists, expanding access to innovative care models, and addressing socioeconomic and geographic disparities are critical to improving outcomes for prostate cancer patients in rural areas. Future research should focus on developing and evaluating these strategies to ensure equitable care for all patients.

## Introduction and background

Prostate cancer is a common malignancy in the prostate gland, mainly affecting men over 65. While rare under 40, its risk increases with age. The disease can significantly impact the quality of life, causing urinary symptoms, sexual dysfunction, and potential metastasis [1]. The incidence of prostate cancer has surged in developed countries, mainly due to the aging population and the advent of improved screening tests such as prostate-specific antigen (PSA) testing [2]. Early diagnosis of prostate cancer is vital, as it affects one in eight men. Screening can reduce mortality by up to 25%, and early-stage detection leads to a nearly 100% five-year survival rate, significantly improving outcomes for older men [3]. Thus, the healthcare system must emphasize effective collaboration between primary care physicians and urologists to provide optimal care for prostate cancer patients [4].

Primary care physicians play a pivotal role in the early detection of prostate cancer. Most initial screenings occur within primary care settings, making these physicians the first line of defense against the disease [5]. When primary care physicians detect potential cases of prostate cancer through routine screenings, they can refer these patients to urologists for further evaluation and management [5]. This collaborative approach ensures that patients receive timely and appropriate care, which is essential for improving health outcomes.

However, in rural areas, several challenges impede effective collaboration between primary care physicians and urologists. The scarcity of urologists and limited knowledge of prostate cancer among primary care physicians can hinder the effective referral and management process [6]. The number of available urologists often depends on the healthcare administration policies of each country, which can vary significantly [7]. Therefore, enhancing the capability of primary care physicians in rural settings to manage prostate cancer is vital for improving patient outcomes.

Investigating the quality of prostate cancer care provided by rural primary care physicians is essential for identifying areas that need improvement. This research aims to evaluate the current state of prostate cancer care in rural primary care settings through a narrative review of academic articles. By compiling and analyzing the existing evidence, we can develop targeted educational interventions to enhance the knowledge and skills of primary care physicians in these areas.

To date, the quality of prostate cancer care in rural primary care settings has not been thoroughly investigated [8]. This gap in research underscores the need for a comprehensive examination of how prostate cancer is managed in rural areas. Understanding the strengths and weaknesses of rural primary care can inform strategies to improve collaboration with urologists and ultimately enhance the quality of care for prostate cancer patients. Early detection and effective disease management rely heavily on collaborating with primary care physicians and urologists. In rural areas, addressing the shortage of urologists and enhancing primary care physicians' knowledge is crucial for improving patient outcomes. This research seeks to explore the quality of prostate cancer care in rural primary care settings, providing a foundation for future educational interventions and policy improvements.

## Review

We performed a narrative review regarding the primary care quality of prostate cancer in rural contexts using PubMed [9]. We searched PubMed for original articles regarding the primary care quality of prostate cancer in rural settings from April 2000 to August 2024. The inclusion criteria were studies focusing on adult male populations, rural healthcare settings, and prostate cancer management, emphasizing primary care interventions and outcomes. We included observational studies, clinical trials, and reviews that discussed healthcare quality or barriers to care in rural areas. Articles were excluded if they primarily addressed urban contexts, focused on secondary or tertiary care, or lacked sufficient data on rural healthcare quality. Regarding articles written in English, our search strategy was based on the following title/abstract keywords: (((prostate cancer) AND (primary care)) AND (management)) AND (rural). The reference lists of relevant studies were also reviewed to identify additional research. In total, 13 articles were included in this review (Table 1).

**Table 1:** Included articles' explanation CC: community care; CI: confidence interval; GP: general practitioner; RR: relative risk; PSA: prostate-specific antigen; USA: United States of America; VCP: Veterans Choice Program

Author	Year	Country	Purpose	Quality of primary care
Campbell NC et al. [10]	2000	Scotland	Evaluate common cancers' quality of care.	Increasing distance from a cancer center was associated with less chance of diagnosis before death for stomach, breast, and colorectal cancers and poorer survival after diagnosis for prostate and lung cancers.
Gormley GJ et al. [11]	2006	Ireland	Examine influences on the behavior of GPs about PSA testing.	Prostate cancer testing is driven by family history, patient requests, and prior asymptomatic case detection. Accredited practices test less. Low guideline awareness indicates a need for better dissemination. Local educational meetings are underutilized.
Jones RA et al. [12]	2011	USA	Explore cancer support and financial issues related to cancer care experienced by African-American men with prostate cancer and understand whom they relied on for resource issues during diagnosis and treatment.	For rural and urban African-American prostate cancer survivors, wives were key support sources. Health insurance reduces anxiety and financial stress. Tailoring cancer care plans to address their specific support needs and challenges is crucial.
Li X et al. [13]	2012	Sweden	Analyze the association between neighborhood deprivation and prostate cancer mortality after adjusting for individual characteristics.	A high level of neighborhood deprivation independently predicts prostate cancer mortality. This raises important clinical and public health concerns. Both individual- and neighborhood-level approaches are important in healthcare policies.
Darwish-Yassine M et al. [14]	2014	USA	Investigate prostate cancer survivors in the Michigan Cancer Registry to identify treatment side effect rates, evaluate access to preventive care services post-treatment, and assess their informational needs regarding prostate cancer.	Persistent symptoms after prostate cancer treatment highlight a gap in symptom management. Long-term studies comparing active surveillance and treatment outcomes are needed. Clinicians should assess ongoing distress, and more resources are needed to educate survivors on their prognosis.
McCombie SP et al. [15]	2015	Australia	Evaluate the structure and outcomes of a new ‘One Stop’ Prostate Clinic (OSPC) designed specifically for rural and remote men.	Among men with a PSA level of 6.7 ng/mL, 92% underwent biopsies, with 60% diagnosed with prostate cancer. Common treatments included radical prostatectomy, active surveillance, and radiation. The OSPC model saved $1045 per person in travel costs.
Pickles K et al. [16]	2015	Australia	Explain GPs’ approaches to PSA testing and overdiagnosis, how they reason about their testing routines, and how these routines impact their personal experiences as clinicians.	GPs’ PSA testing practices vary based on their views on overdiagnosis and underdiagnosis, leading to differing care for men. Addressing overdiagnosis in policy should consider these variations in GP practices.
Nikolaidis C et al. [17]	2015	Greece	Identify higher cancer mortality rates, particularly for digestive and prostate cancers, in rural versus urban areas of north-eastern Greece.	A more pronounced discrepancy was observed for prostate cancer mortality (RR=1.86, 95%CI=1.10–3.14), indicating a strong positive correlation with rurality.
Cary C et al. [18]	2016	USA	Assess variation in the primary treatment of prostate cancer by examining the effect of population density of the county of residence on treatment for clinically localized prostate cancer and quantify variation in primary treatment attributable to the county and state level.	Men with localized prostate cancer in metropolitan counties had 23% higher odds of receiving surgery or radiation compared to rural men. County of residence accounted for 6% of treatment variation.
Wills MJ et al. [19]	2017	USA	Examine travel distances for rural Medicare beneficiaries seeking treatment for prostate, breast, or colorectal cancer in the Deep South.	Healthcare changes, like hospital closures and consolidations, increase travel distances for cancer treatment, particularly in the Deep South. This highlights the need to address disparities in rural health and improve access to nearby treatment facilities.
Ivers R et al. [20]	2019	Australia	Evaluate the acceptability of a cancer care team based at an Australian Aboriginal medical service in supporting patients' cancer journeys and assess improvements in access to cancer care.	A primary care-based cancer care team in an Australian Aboriginal medical service offered culturally safe, accessible care with home visits and transport, improving client access to necessary services.
Erickson BA et al. [21]	2023	USA	Describe the prevalence and patterns in VCP-funded purchased CC after the implementation of the VCP among veterans with prostate cancer.	The percentage of veterans receiving definitive treatment in VCP-funded purchased care settings increased significantly, but this increased access may lead to overtreatment and lower care quality for low-risk prostate cancer.
Dwyer ER et al. [22]	2023	Australia	Evaluate telehealth's impact on satisfaction and costs for rural urologic cancer patients.	Rural patients in telehealth groups were more likely than urban patients to prefer future telehealth appointments (67% vs. 58%). Rural in-person appointments had a higher financial burden compared to telehealth (medians, $80 vs. $0).

**Figure 1:**
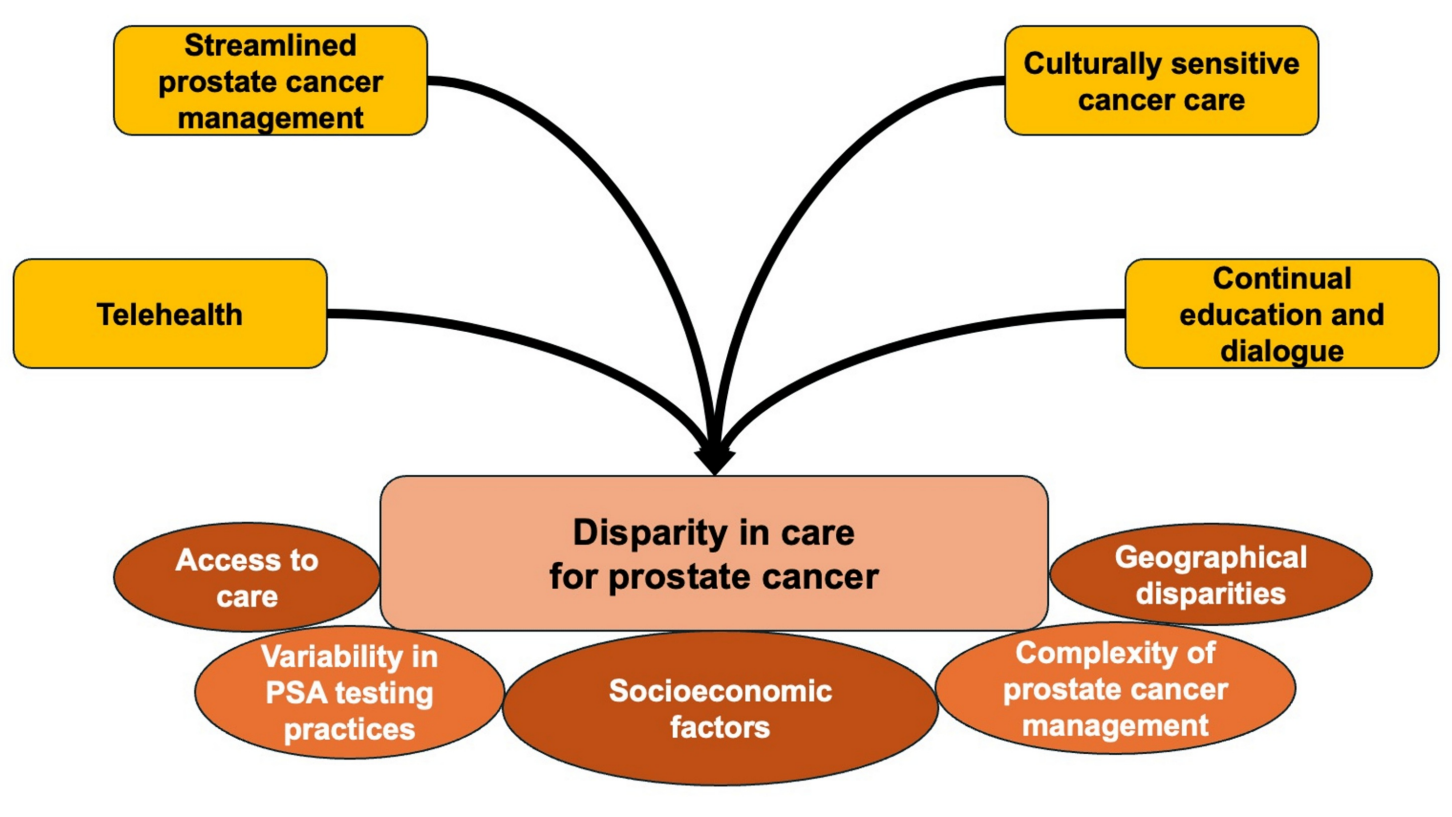
A concept figure of challenges and solutions for prostate cancer care in rural primary care PSA: prostate-specific antigen Image credit: Ryuichi Ohta

Reading the included articles, we summarized the primary care quality of prostate cancer in rural contexts. We discussed possible action plans to improve the primary care quality of prostate cancer in rural contexts (Figure 1).

Difficulty in approaching effective management of prostate cancer in primary care

The reviewed studies underscore significant challenges and disparities in managing prostate cancer, focusing on access to care, support systems, and socioeconomic factors. Jones RA (2011) conducted a qualitative study in the USA to explore the cancer support and financial issues faced by African-American men with prostate cancer. The study found that both rural and urban African-American survivors relied heavily on their wives for support. Health insurance is critical in reducing anxiety and financial hardships [12]. These insights highlight healthcare providers' need to comprehend and address this demographic's specific support needs and challenges to create more effective cancer health plans. Erickson BA (2023) conducted a retrospective cohort study in the USA examining the prevalence and patterns of prostate cancer care funded by the Veterans Choice Program (VCP) among veterans [21]. The study noted a significant rise in veterans receiving definitive treatment throughout the study period. Despite this increased access, the findings suggest a potential trade-off between access and care quality, with concerns over the overtreatment of low-risk prostate cancer cases. This highlights the possibility that expanded access may lead to compromised quality, particularly in managing low-risk patients effectively.

Living conditions and supporting systems affected prostate cancer management. Li X (2012) analyzed the association between neighborhood deprivation and prostate cancer mortality in Sweden through a prospective cohort study [13]. The study concluded that high levels of neighborhood deprivation independently predicted higher prostate cancer mortality, raising significant clinical and public health concerns. This finding underscores the need for healthcare policies to address individual and neighborhood-level factors to mitigate such disparities. Wills MJ (2017) examined travel distances for rural Medicare beneficiaries in the USA seeking treatment for prostate, breast, or colorectal cancer [19]. The study found that hospital closures and consolidations forced patients to travel greater distances for care, exacerbating the lack of access to treatment facilities. The findings emphasize the need for targeted efforts to address healthcare access disparities in rural areas.

The disparity between rural and urban conditions significantly impacts prostate cancer care. Campbell NC et al. (2000) conducted a retrospective cohort study in Scotland, revealing that increasing distance from a cancer center was associated with poorer survival rates after diagnosis of prostate cancer [10]. This finding underscores the critical role that proximity to treatment facilities plays in patient outcomes, with rural patients facing a heightened risk of worse prognoses due to reduced access to timely and effective cancer care. The study’s results emphasize the need for improved healthcare access in rural areas to enhance prostate cancer outcomes. Nikolaidis C (2015) conducted a case study in Greece to investigate regional disparities in cancer mortality [17]. The study found a pronounced discrepancy in prostate cancer mortality, strongly correlated with rurality. This finding indicates that patients in rural areas face significantly higher mortality risks, underscoring the need for more focused healthcare interventions in these regions. These studies collectively highlight the complex interplay of support systems, healthcare access, socioeconomic factors, and geographical disparities in managing prostate cancer. Addressing these challenges requires multifaceted approaches tailored to the unique needs of different populations to improve care quality and patient outcomes.

Management challenges among primary care physicians

The included studies focus on the management challenges primary care physicians face in prostate cancer care, highlighting variations in practices and the impact of geographic and socioeconomic factors. Understanding PSA for the diagnosis of prostatic cancer is challenging among primary care physicians. Gormley GJ (2006) conducted a cross-sectional study in Ireland to identify factors influencing PSA testing behavior among primary care physicians [11]. Key influences included testing men with a positive family history, unrelated complaints, patient requests, and previous experiences of detecting prostate cancer in asymptomatic patients via PSA testing. Lower testing levels were observed in accredited training practices. The study highlights a low awareness of national guidelines and emphasizes the untapped potential of local educational meetings to influence PSA testing behavior. This underscores the need for new strategies to disseminate and implement these guidelines effectively. Pickles K (2015) explored the varied approaches of Australian general practitioners (GPs) to PSA testing and the issue of overdiagnosis through a qualitative study [16]. The findings revealed significant variability in GPs’ PSA testing practices, influenced by their views on overdiagnosis and underdiagnosis of prostate cancer. This variability resulted in men receiving different levels of care depending on their GP’s reasoning and practice preferences. The study suggests that future policies addressing overdiagnosis need to consider these patterned variations in practice to be effective.

The treatments and follow-up of prostate cancer in primary care are controversial and diverse. Darwish-Yassine M (2014) conducted a cross-sectional survey in the USA to evaluate long-term outcomes in prostate cancer survivors [14]. The study identified persistent symptoms post-treatment, indicating a gap in symptom management. It advocates for long-term research comparing active surveillance and active treatment to minimize the burden of long-term physical symptoms. The study emphasizes the need for clinicians to assess post-treatment distress and provide ongoing supportive care.

Additionally, it calls for more informational resources to educate prostate cancer survivors about their prognosis and post-treatment care. Cary C (2016) also conducted a cross-sectional study in the USA to assess the impact of population density on prostate cancer treatment [18]. The study found that men with localized prostate cancer in metropolitan counties had a 23% higher likelihood of being treated with surgery or radiation compared to those in rural counties, even after controlling for the number of urologists per county and other clinical and sociodemographic characteristics. The study quantified that 3% of treatment variation was attributable to the Surveillance, Epidemiology, and End Results (SEER) site, while 6% was due to the county of residence. These findings highlight significant disparities in prostate cancer treatment based on geographic location, emphasizing the need for targeted efforts to address these disparities. These studies underscore the complex interplay of individual physician practices, geographic factors, and patient education in effectively managing prostate cancer. Addressing these challenges requires multifaceted approaches, including better guideline dissemination, educational interventions, and policies to reduce geographic disparities. Such efforts are essential to enhance the quality of care and ensure equitable treatment outcomes for all prostate cancer patients.

Effective collaboration with oncologists and general hospital in-person and via online communication

The included studies explore innovative approaches to cancer care delivery for rural and underserved populations, emphasizing the importance of effective collaboration between oncologists, general hospitals, and primary care settings through in-person and online communication.

One of the solutions for the effective management of prostate cancer is suggested to be telecommunication. Dwyer ER (2023) conducted a cross-sectional study in Australia to assess the effectiveness of telehealth for rural patients with urologic cancer [22]. The study found that 67% of rural patients strongly preferred telehealth for future appointments, compared to 58% of urban patients, primarily due to lower cost and time commitments. Rural patients attending in-person appointments faced significantly higher financial burdens (median cost $80) than those using telehealth (median cost $0). These findings underscore telehealth's potential to alleviate financial strain and improve access to care for rural patients, making it a favorable alternative to traditional in-person visits in primary care.

Another solution is the effective collaboration among primary care physicians and centers for smooth consultation and treatment flow of prostate cancer. McCombie SP (2015) evaluated a 'One Stop' Prostate Clinic (OSPC) in Australia designed specifically for rural and remote men [15]. This prospective cohort study reported an average PSA level of 6.7 ng/mL among patients, with 39% exhibiting suspicious digital rectal examination (DRE) results. Of the 200 men, 92% underwent prostate biopsies, and 60% were diagnosed with prostate cancer. Common treatments included radical prostatectomy (41%), active surveillance (25%), and external beam radiation therapy (23%). The OSPC model demonstrated substantial cost savings, estimated at $1045 per patient, by reducing the need for travel. The clinic's streamlined approach facilitated timely diagnosis and treatment, effectively addressing the healthcare needs of rural men.

For the effective implementation of novel management methods of prostate cancer in primary care, respect for culture and society in communities was essential. Ivers R (2019) conducted a qualitative study evaluating a cancer care team integrated into an Australian Aboriginal medical service [20]. The team significantly improved accessibility to cancer care by providing home visits, transportation, and accompaniment to tertiary care settings. The service was perceived as culturally safe and accessible, enhancing its acceptability among Aboriginal patients. This model of care addressed the unique cultural and logistical needs of Aboriginal communities, ensuring that patients received timely and appropriate cancer care.

These studies highlight the effectiveness and acceptability of innovative cancer care models tailored for rural and underserved populations. Telehealth has proven to be a cost-effective and preferred option for rural patients, reducing financial burdens and improving access to care. The OSPC model offers significant cost savings and efficient cancer diagnosis and treatment for rural men. The culturally sensitive cancer care team has also enhanced service accessibility and acceptability among Australian Aboriginal patients. Collectively, these approaches underscore the need for tailored healthcare strategies that address the specific challenges faced by underserved populations, ultimately improving cancer care outcomes and patient satisfaction.

Discussion

The findings of our narrative review underscore the significant disparities and challenges in managing prostate cancer in rural primary care settings. The reviewed studies highlight several critical areas where improvements can be made to enhance the quality of care for prostate cancer patients.

One of the primary challenges identified is the limited access to urologists in rural areas. This scarcity often results in delayed diagnosis and treatment, adversely impacting patient outcomes. The study by Erickson et al. (2023) illustrates how increased access to care, while beneficial, can also lead to overtreatment, particularly for low-risk prostate cancer patients [21]. This highlights the delicate balance between ensuring timely access and avoiding unnecessary interventions. Previous studies also show the gap in access to cancer treatment between rural and urban regions impinges on the morbidity and mortality of patients [6,23]. To effectively manage cancer treatment in primary care, general oncologists should be allocated in rural contexts and collaborate with primary care physicians and citizens to improve the quality of care. 

Socioeconomic factors and support systems play a crucial role in patient outcomes. The study by Jones et al. (2011) reveals that African-American men, particularly those in rural areas, rely heavily on personal support systems and face significant financial burdens [12]. This underscores the need for healthcare providers to consider these factors when planning and delivering care. Rural areas especially lack medical resources and support for isolated people, including cancer patients [19,24]. Such situations influence their medical care regarding prostate cancer treatments. In addition, symptoms regarding urinary tracts are challenging to confess among older male patients, which can delay the detection of prostate cancers [25]. Policies to reduce these burdens and provide robust support systems can significantly improve patient experiences and outcomes. Their help-seeking behaviors should also be revised through the provision of information and education for alarming symptoms in urinary tracts showing prostate cancer [26].

Geographical disparities also significantly affect prostate cancer management. Studies such as those by Li et al. (2012) and Campbell et al. (2000) demonstrate the negative impact of living in deprived neighborhoods or far from treatment centers on prostate cancer mortality [10,13]. These findings suggest that healthcare policies must address individual and neighborhood-level factors to mitigate these disparities effectively. In rural contexts, isolation and loneliness are critical issues, increasing morbidity and mortality [27]. The effects may apply to prostate cancer situations. Increasing citizens’ community participation and dialogue is essential to improving social isolation and loneliness [28]. Primary care physicians should have dialogues with people in communities and collaborate with them to increase dialogue regarding health issues.

The variability in PSA testing practices among primary care physicians, as highlighted by Gormley et al. (2006) and Pickles et al. (2015), points to a lack of consensus and standardized guidelines in rural settings [11,16]. This variability can lead to inconsistent care and potential overdiagnosis or underdiagnosis. There is a clear need for better dissemination and implementation of national guidelines to ensure uniformity in practice. In education for primary care physicians, cancer education is lacking, and they do not have enough confidence in the effective detection and reference of prostate cancer [29]. Primary care physicians may be reluctant to refer their patients to urologists because of ineffective collaboration [29]. For the effective detection of prostate cancer, in addition to educating citizens, primary care physicians should be educated, especially about the usage of PSA, and establish mutual understanding and collaboration methods with urologists in hospitals supported by local governments.

The reviewed studies also emphasize the importance of long-term follow-up and symptom management in prostate cancer care. Darwish-Yassine et al. (2014) highlight the persistent symptoms post-treatment and the need for ongoing supportive care [14]. This calls for comprehensive survivorship care plans that address recovery's physical and psychological aspects. Besides, innovative approaches to care delivery, such as telehealth and OSPCs, show promise in addressing some of these challenges. Dwyer et al. (2023) found that telehealth significantly reduced financial burdens for rural patients, making it a viable alternative to in-person visits through collaboration among primary care physicians and specialists [22].

Similarly, the OSPC model, as evaluated by McCombie et al. (2015), demonstrated substantial cost savings and efficient care delivery through continual implementation based on the understanding of primary care physicians and patients [15]. In rural primary care, effective collaboration among primary care physicians, urologists, other medical professionals, and citizens is essential, and continual communication and dialogue among them can develop new innovative approaches [30]. To continue such activities in the long term, maintaining and improving their relationship is critical, leading to effective revision of the activities.

This study has several limitations. Firstly, the narrative review methodology may introduce bias, as the selection of studies and interpretation of findings can be subjective. Additionally, relying on PubMed as the sole database may have excluded relevant studies from other databases. The variability in study designs, populations, and healthcare systems across the included studies also limits the generalizability of our findings. Moreover, the reviewed studies predominantly focus on high-income countries, which may not represent rural settings in low- and middle-income countries. Future research should aim to include a broader range of databases and consider more diverse geographical settings to provide a more comprehensive understanding of the issues at hand.

Future directions

Future research should focus on expanding the scope of studies to include rural settings in low- and middle-income countries, where the challenges in prostate cancer management may differ significantly from those in high-income regions. Additionally, there is a need for more robust, standardized guidelines for PSA testing and prostate cancer management in rural primary care. Collaborative efforts between local governments, primary care physicians, and specialists should be emphasized to develop tailored interventions that address the unique needs of rural populations. Longitudinal studies are also essential to assess the long-term impact of innovative models like telehealth and OSPCs on patient outcomes and healthcare equity.

## Conclusions

Our narrative review highlights significant challenges and disparities in the management of prostate cancer in rural primary care settings. Key issues include limited urologist access, socioeconomic and geographical disparities, variability in PSA testing practices, and the need for comprehensive long-term care. Innovative care models such as telehealth and OSPCs show the potential to address these challenges. To improve prostate cancer care in rural areas, there is a need for targeted educational interventions, better dissemination of guidelines, and policies that address both individual and neighborhood-level factors. Future research should focus on developing and evaluating tailored healthcare strategies that can enhance the quality of care and ensure equitable treatment outcomes for all prostate cancer patients.
